# Cartilage Regeneration in Human with Adipose Tissue-Derived Stem Cells: Current Status in Clinical Implications

**DOI:** 10.1155/2016/4702674

**Published:** 2016-01-06

**Authors:** Jaewoo Pak, Jung Hun Lee, Wiwi Andralia Kartolo, Sang Hee Lee

**Affiliations:** ^1^Stems Medical Clinic, 32-3 Chungdam-dong, Gangnam-gu, Seoul 06068, Republic of Korea; ^2^National Leading Research Laboratory, Department of Biological Sciences, Myongji University, 116 Myongjiro, Yongin, Gyeonggido 17058, Republic of Korea; ^3^FMN Wellness & Antiaging Centre, Jalan Sangihe 15A, Jakarta Pusat 10150, Indonesia

## Abstract

Osteoarthritis (OA) is one of the most common debilitating disorders among the elderly population. At present, there is no definite cure for the underlying causes of OA. However, adipose tissue-derived stem cells (ADSCs) in the form of stromal vascular fraction (SVF) may offer an alternative at this time. ADSCs are one type of mesenchymal stem cells that have been utilized and have demonstrated an ability to regenerate cartilage. ADSCs have been shown to regenerate cartilage in a variety of animal models also. Non-culture-expanded ADSCs, in the form of SVF along with platelet rich plasma (PRP), have recently been used in humans to treat OA and other cartilage abnormalities. These ADSCs have demonstrated effectiveness without any serious side effects. However, due to regulatory issues, only ADSCs in the form of SVF are currently allowed for clinical uses in humans. Culture-expanded ADSCs, although more convenient, require clinical trials for a regulatory approval prior to uses in clinical settings. Here we present a systematic review of currently available clinical studies involving ADSCs in the form of SVF and in the culture-expanded form, with or without PRP, highlighting the clinical effectiveness and safety in treating OA.

## 1. Introduction

Osteoarthritis (OA) is a common painful and debilitating disorder in the elderly [1, 2]. All current medical treatments for OA, such as nonsteroidal anti-inflammatory drugs (NSAIDs), steroids, and hyaluronic acids (HAs), physical therapy, aim to remedy the symptoms, as opposed to treating the underlying causes. When failed with symptomatic medical treatments, patients usually resort to receiving total knee replacement (TKR) or total hip replacement (THR) surgery. Both TKR and THR surgeries carry relatively high morbidity and mortality rates [1, 2]. Even with improved surgical technique, anesthesia, and rehabilitation, the thirty-day mortality rate after total knee arthroplasty is reported to be 0.18%, and 5.6% of the patients experienced complications [3]. Also, the overall 30- and 90-day mortality rates for total hip arthroplasty are reported to be 0.24% and 0.55%, respectively [4]. These approaches do not address the morbidity associated with early disease or the limitations of arthroplasty surgery, which include the possibility of adverse outcomes and the finite lifespan of prostheses [5].

Mesenchymal stem cells (MSCs) are found in numerous human tissues including bone marrow and adipose tissue [6, 7]. These MSCs have been shown to differentiate into bones, cartilage, muscle, and adipose tissue [6–8]. Because of their potential capabilities in regenerating cartilage, MSCs have been successfully used in animals [9, 10]. In 2008, Centeno et al. have showed successful cartilage regeneration in humans with MSCs [11]. Subsequently, in 2010, the same group also reported safety data of using MSCs in humans for cartilage regeneration [12].

Adipose tissue-derived stem cells (ADSCs) are one type of MSCs. In 2001 and 2002, Zuk et al. showed that adipose tissue in the form of stromal vascular fraction (SVF) contains stem cells that have the capacity to differentiate into cartilage, bone, muscle, and adipose tissue, similar to MSCs [13, 14]. Likewise, ADSCs also have been investigated in treatment of cartilage injuries and osteoarthritis in animals. The results from these studies showed evidence of cartilage regeneration by using ADSCs [15–19].

Consequently, in 2011, Pak successfully treated 2 human patients with OA of the knees by using autologous ADSCs in the form of SVF along with platelet rich plasma (PRP) and hyaluronic acid (HA). He documented the regeneration of cartilage-like tissue in these patients through magnetic resonance imaging (MRI) studies [20].

More studies have recently become available, providing more evidence of cartilage regeneration in human patients with OA of the knees [21–23]. Such continued research and interests hold great promises in the field of regenerative medicine.

Although the successful regeneration of cartilage with ADSCs in humans may represent a promising, minimally invasive, nonsurgical alternative, many issues need to be resolved and clarified before the general application of this procedure. The mechanism of regeneration remains unclear: (i) it could be due to the secretory effects of the stem cells injected [24, 25]; (ii) it could be due to direct engraftment and differentiation of the stem cells that were introduced into the diseased joints [26, 27]; or (iii) it could be due to the combination of secretory effects and direct engraftment of the stem cells.

Adipose stem cells excrete a variety of cytokines, chemokines, growth factors, and exosomes [28, 29]. These factors have positive effects on the surrounding progenitor cells. However, there is some evidence that these stem cells injected may actually become engrafted into the tissue and differentiate into tissue-specific stem cells [30]. It is also very possible that these two mechanisms play a role in cartilage regeneration.

Furthermore, the method of the cell transplant needs to be studied in detail: the most optimal dosage of the stem cells to be injected, the best mode of injection, the best method of promoting stem cell adherence to the lesions, and the most potential growth factors (e.g., PRP) to be added, as well as the best scaffolding materials (e.g., HA and extracellular matrix (ECM)).

Here we will present a comprehensive and systematic review of cartilage regeneration in human joints by using ADSCs in the form of adipose SVF and assess the possibility of the clinical application of these stem cells.

## 2. Method 

**Figure 1:**
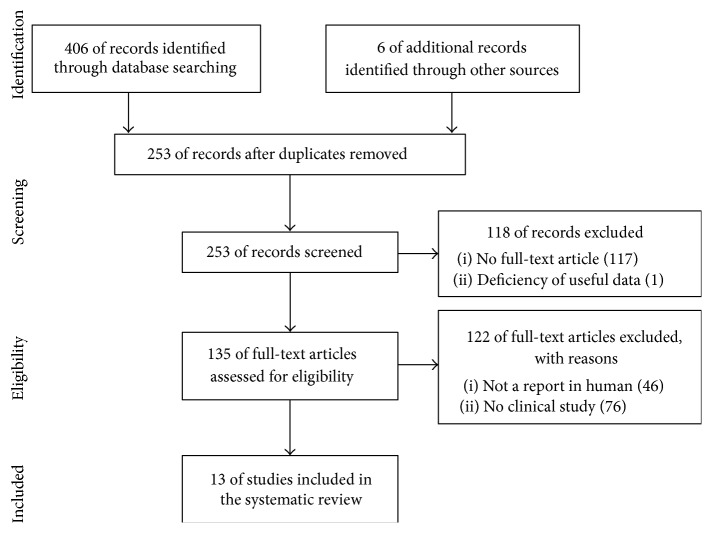
Literature selection process (PRISMA flow diagram).

We used the Preferred Reporting Items for Systematic Review and Meta-Analysis (PRISMA) in our review (Figure 1) [31]. We conducted a systematic literature search in PubMed, Medline, and Embase. We used the keywords as our search terms. We combined terms for selected indications (stem cell, osteoarthritis, and adipose). The literature search included all studies published in English between 2000 and 2015. We identified 253 references after removing duplicates. We independently assessed full-text articles for inclusion in our review. The criteria for the inclusion of studies in our review encompassed clinical studies on ADSC injection conducted on humans for cartilage regeneration. Finally, we found 13 articles showing clinical studies on ADSC treatments for cartilage defects (Figure 1).

## 3. ADSCs in the Form of SVF along with PRP and/or HA/ECM

At present, most of the ADSCs being used in clinical settings are in the form of SVF. To obtain adipose SVF, liposuction is performed on easily accessible areas of the body, such as the abdomen, buttocks, or thighs. These lipoaspirates are then digested with collagenase to extract stem cells that exist within the matrix of the adipose tissue [13, 14]. The collagenase is then washed off using a centrifuge and dilution method. The pellet, including the bottom portion of the centrifuge, is considered to be SVF [13, 14]. SVF contains a variety of cells in different proportions: ADSCs, a type of mesenchymal stem cells, pericytes, vascular adventitia cells, fibroblasts, preadipocytes, monocytes, macrophages, red blood cells, fibrous tissue, ECM, and so forth [13, 14].

The process of preparing adipose SVF is considered to be a medical procedure in Korea and a few other countries when performed by a physician as a single surgical procedure within the same day and with minimal manipulations [32]. Unlike adipose SVF, culture-expanded stem cells are usually considered to be pharmaceutical products, requiring clinical trials and governmental approval.

### 3.1. Number of Stem Cells in Human Adipose Tissue

The number of stem cells that can be extracted from each individual varies greatly.

Currently, it is well accepted that ADSCs exist within the matrix of adipose tissue. More specifically, it has been shown that ADSCs exist around blood vessels of adipose matrix [33]. These stem cells can be released from the matrix by processing the lipoaspirate with collagenase. Such stem cells are shown to regenerated cartilage as shown by Zuk et al. [13, 14]. However, the number of stem cells that can be extracted from one gram of adipose tissue can be very variable in different individual patients [13, 34–39].

The number of stem cells that can be obtained from one gram of adipose tissue can range from 5,000 to 200,000 cells [40], which have been measured by flow cytometry and indirect immunofluorescence [41, 42]. Such large individual variability may result in inconsistency of results in treating patients. Patients with high number of stem cells will have great cartilage regeneration. However, patients with low number of stem cells will not a great response, as shown by Jo et al. [21].

### 3.2. Autologous Platelet Rich Plasma (PRP)

Autologous PRP was used in most of 13 articles showing clinical studies on ADSC treatments for cartilage defects.

PRP contains a variety of growth factors: transforming growth factor-*β* (TGF-*β*), epidermal growth factor (EGF), and fibroblast growth factor (FGF), along others [43]. These growth factors are known to proliferate stem cells. Centeno et al. used autologous platelet lysate to grow bone marrow-derived stem cells, which were injected in human patients for cartilage regeneration [11]. Likewise, PRP has been used to increase the number of stem cells injected into a joint.

Also, activated PRP may act like a scaffold for stem cells. Autologous PRP has been prepared by centrifuging autologous blood with anticoagulant citrate dextrose solution [11, 44]. When autologous PRP has been activated by adding calcium chloride, thrombin, or collagen [11, 44–46], PRP may become a “curd-like” substance [11], which may function like a scaffold.

### 3.3. Hyaluronic Acid (HA) and Extracellular Matrix (ECM)

Scaffolding materials were used in some [20, 47–49] of the 13 articles showing clinical studies on ADSC treatments for cartilage defects.

HA and ECM are two naturally occurring scaffolding materials. HA has a high affinity for cartilage defects and provides an environment for stem cells to adhere to the lesion and differentiate [50]. ECM also provides an environment for stem cells to adhere and differentiate [51]. When autologous ECM is provided, immune reactions are not likely to occur. In addition, ECM contains a variety of growth factors, which further enhance the growth and differentiation of the injected stem cells [51].

### 3.4. ADSCs with PRP and/or HA/ECM

The combination of ADSCS with PRP and/or scaffolding materials was used in 13 articles showing clinical studies on ADSC treatments for cartilage defects.

PRP or platelet lysate provides a variety of growth factors for stem cells [11, 43]. HA/ECM scaffolding materials provide the environment for stem cells to adhere and differentiate into cartilage [50, 51]. Together, this combination may provide the best optimal strategy for stem cells to adhere, grow, and differentiate into cartilage [20, 22, 44, 47–49, 52–55].

## 4. Clinical Applications of ADSCs 

The main features of clinical studies on ADSC treatments for cartilage defects were summarized in Table 1.

### 4.1. Case Report by Pak [20]

This is the very first study that showed the possibility of ADSCs in the form of SVF regenerating cartilage in human patients. Pak used approximately 100 g of adipose tissue obtained from the abdomen. This adipose tissue was digested with collagenase. The collagenase was washed off. The resulting adipose SVF, containing ADSCs, was injected percutaneously with calcium chloride-activated PRP, HA, and dexamethasone into joints of 2 patients with OA. Three months after the injections, the visual analog score (VAS) for pain, functional rating index, and range of motion (ROM) improved along with the MRI evidence of cartilage-like tissue regeneration in these patients.

This study used 100 g of adipose tissue. Thus, the total estimated number of ADSCs injected can range from 500,000 to 20,000,000 [40]. Also, it should be noted that this study used PRP and HA, along with ADSCs.

### 4.2. Nonrandomized, Retrospective, and Comparative Study by Koh and Choi [52]

This study involved 25 patients with OA of the knees. The patients were injected with adipose SVF derived from approximately 19 g of adipose tissue obtained from the knee fat pad while performing arthroscopic lavage and debridement. Thereafter, the adipose SVF was percutaneously injected with calcium chloride-activated PRP. A mean of 1.89 × 10^6^ ADSCs was presented in 19 g of adipose SVF. The results showed that the mean Lysholm knee scoring scales, Tegner activity scales, and VAS scores in the study group had improved significantly compared to the control group. No major adverse events were observed.

In this study, the approximate number of ADSCs obtained was little less than 2,000,000, and this was calculated to be little less than 100,000 stem cells per gram of adipose tissue. The study concludes that little less than 2 million of ADSCs with PRP were effective.

### 4.3. Retrospective Cohort Study by Pak et al. [44]

This is the very first safety report involving human ADSCs in the form of SVF. Between the period of 2009 and 2010, Pak et al. injected joints percutaneously with the autologous, non-culture-expanded ADSCs in 91 patients. In 2013, Pak et al. reported that all 91 patients had no serious side effects and no cancer was reported. However, the study reported that a few minor side effects occurred, mainly swelling and tendonitis, both of which were ameliorated with NSAIDs. The average efficacy reported was 65% at 3 months after the treatment.

All these patients were injected with approximately 100 g of adipose tissue. Thus, the total estimated number of ADSCs injected can range from 500,000 to 20,000,000 [40].

### 4.4. Case Series by Pak et al. [47]

This study involved 3 patients with chondromalacia patellae of the knees. The patients were treated with ADSCs in the form of SVF, calcium chloride-activated PRP, and HA. The mixture was injected into the knees percutaneously. After 3 months of the treatment, the patients' VAS pain scale, functional rating index (FRI), and ROM had improved. The study also showed positive regeneration of hyaline cartilage-like tissue at the patellofemoral joints of all 3 patients.

This is the very first study showing the possibility of treating chondromalacia patellae with ADSCs with PRP and HA.

### 4.5. Case Series by Koh et al. [53]

This study involved 18 patients with OA of the knees. The patients received non-culture-expanded ADSCs in the form of SVF obtained from the knee fat pad. The ADSCs were percutaneously injected into the knees with calcium chloride-activated PRP after arthroscopic debridement of the knees. A mean of 1.18 × 10^6^ ADSCs was prepared from approximately 9.1 g of adipose tissue from the knee fat pad. Thereafter, Western Ontario and McMaster Universities osteoarthritis index (WOMAC), Lysholm, and VAS scores were measured and improved. The whole-organ MRI score, particularly the cartilage whole-organ MRI score, also improved. The authors concluded that improvements in the clinical and MRI results were positively related to the number of ADSCs injected.

This study used little over one million ADSCs obtained from mean of 9.1 g of adipose tissue obtained from the knee fat pad along with PRP. The number of ADSCs extracted from 1 g of adipose tissue was approximately 129,700 ADSCs per gram of adipose tissue.

### 4.6. Case Series by Koh et al. [22]

This study involved 30 patients with OA of the knees. The patients were injected with adipose SVF containing ADSCs extracted from 120 g of adipose tissue from the buttocks. The adipose SVF were injected with calcium chloride-activated PRP under arthroscopic guidance after arthroscopic lavage. Of these patients, 16 patients went through the second-look arthroscopies in a median of 25 months after the initial treatment. At a minimum of 2 years after the operation, almost all patients showed significant improvement in the knee injury, OA outcome scores (KOOS), VAS pain scale, and Lysholm score. In the second-look arthroscopy, 10 patients (63%) had improved cartilage, 4 patients (25%) had maintained the cartilage, and 2 patients (12%) failed in healing cartilage defects.

This study used 120 g of adipose tissue from buttock. Unlike other previous reports, the study reported extracting only little over 4 million ADSCs from 120 g of adipose tissue. However, this study is the very first study showing direct evidence of cartilage regeneration via arthroscope.

### 4.7. Case Report by Pak et al. [48]

This study involved 1 patient with a meniscus tear of the knee. The patient was treated with autologous adipose SVF containing ADSCs derived from approximately 40 g of packed adipose tissue obtained from the abdomen. The adipose SVF was injected with calcium chloride-activated PRP and HA. After 3 months, the patient's VAS for pain, FRI, and ROM had improved. Furthermore, the meniscus tear had improved, if not entirely disappeared, in the subsequent follow-up MRIs after 3 months.

This is another first case report showing the possibility of treating meniscus tear with ADSCs with PRP and HA.

### 4.8. Case Series by Bui et al. [54]

This study involved 21 patients with OA of the knees with grades 2 and 3. The patients were treated with autologous ADSCs in the form of SVF obtained from the abdomen. The ADSCs were injected percutaneously into the joints with calcium chloride-activated PRP. All 21 patients showed improved joint function after 8.5 months, measured by VAS pain score and the Lysholm score. In addition, significant improvements were noted in the MRI findings with increased thickness of the cartilage layer.

This study used 50–100 g of lipoaspirates. Thus, the number of ADSCs injected may range from 250,000 to 20,000,000. All these ADSCs were injected with PRP with good response.

### 4.9. Double-Blind, Randomized Dose Escalation Study by Jo et al. [21]

This is the very first double-blind, randomized clinical trial involving ADSCs in 18 patients. The patients received autologous culture-expanded ADSCs via arthroscopy. No arthroscopic lavage was performed and no PRP was injected. The ADSCs suspended in 3 mL of normal saline were injected. Initially, there were 3 groups: low-dose (1.0 × 10^7^ ADSCs), mid-dose (5.0 × 10^7^ ADSCs), and high-dose (1.0 × 10^8^ ADSCs) groups with 3 patients each. In the high-dose group, there was a significantly increased volume of cartilage regeneration compared to mid-dose and low-dose group. The regeneration of the cartilage was confirmed by MRI and arthroscopy. Furthermore, the histology of the regenerated tissue was consistent with hyaline cartilage in characteristics. After such results in the first 9 patients, the remaining 9 of the 18 patients received high-dose (1.0 × 10^8^) ADSCs. There were no treatment-related adverse events and the WOMAC score improved.

This is the very first double-blind, randomized study with 3 different dosages of ADSCs. Unlike other studies, Jo et al. used only autologous culture-expanded ADSCs without PRP and without HA. This study clearly shows that ADSCs are effective in regenerating cartilage. This study also showed that higher dosage of ADSCs (100 million) is more efficacious than lower number of ADSCs (10 million).

### 4.10. Case Series by Koh et al. [23]

This is a second-look arthroscopic study involving 35 patients with a total of 37 knee joints with OA. The patients were treated with ADSCs contained in SVF obtained from a mean of 22.6 g of fat originating from the buttocks. The mean ADSCs obtained from SVF were 3.83 × 10^6^. The ADSCs were injected with calcium chloride-activated PRP under arthroscopic guidance after arthroscopic lavage.

After the mean follow-up period of 12.7 months, second-look arthroscopy was performed. The mean International Knee Documentation Committee (IKDC) and Tegner activity scale scores significantly improved in 94% of the patients. However, 76% of the patients had abnormal repair tissue at second-look arthroscopies. The authors concluded that a scaffolding material may be needed for large lesions.

This study used little less than 4 million ADSCs obtained from 22.6 g of adipose tissue from buttocks. Although PRP was injected with ADSCs, some of the patients did not respond well, necessitating a scaffolding material for better results.

### 4.11. Comparative Study by Koh et al. [55]

This study involved 44 patients and compared the clinical results and second-look arthroscopic findings of a PRP-only treatment group and ADSCs in the form of SVF with a PRP treatment group. Both groups underwent open-wedge high tibial osteotomies (HTO). ADSCs were obtained from 120 g of adipose tissue and injected with PRP in 23 patients. The other 21 patients who went through HTO were injected with PRP only. After following the patients for 24 months, the ADSC with PRP group showed significantly greater improvement in the VAS for pain and KOOS subscales for pain and symptoms, compared to the PRP-only group. However, the Lysholm score was similarly improved in both groups. Arthroscopic evaluation showed that fibrocartilage was regenerated in 50% of the ADSCs with PRP group. Only 10% in the PRP-only group had their fibrous cartilage regenerated. The authors concluded that ADSCs with PRP are more effective than PRP alone.

This study used 120 g of adipose tissue. Thus, the number of ADSCs injected may range from 600,000 to 24,000,000 cells. This study also showed that ADSCs with PRP are more effective than PRP alone.

### 4.12. Comparative Study by Kim et al. [49]

This study involved 54 patients with a total of 56 affected knees in comparing the efficacy of ADSCs in the form of SVF-only group to that of ADSCs-with-fibrin-glue group. The fibrin glue was used as a scaffold. Adipose SVF were obtained from 120 g of adipose tissue. A total of 37 patients (39 knees) were treated with ADSCs only, and the other 17 patients were injected with ADSCs with fibrin glue. After a mean follow-up period of 28.6 months, the mean IKDC score and Tegner activity scale in both the groups significantly improved. However, better International Cartilage Repair Society (ICRS) scores were achieved in the ADSCs-with-fibrin-glue group in the second-look arthroscopies.

This study used 120 g of adipose tissue in comparing ADSCs versus ADSCs with fibrin glue as a scaffold. As expected, ADSCs-with-fibrin-glue scaffold were more effective.

### 4.13. Multicenter Case Control Study by Michalek et al. [56]

This study involved 1,114 patients with OA of the knee and hip from the USA, Czech Republic, Slovakia, and Lithuania. The patients were percutaneously injected with ADSCs in the form of SVF obtained from 20–90 g of adipose tissue. These patients were then followed up for a median of 17.2 months. The clinical effects were measured on the basis of pain, nonsteroid analgesic usage, limping, extent of joint movement, and stiffness. There were no serious side effects reported, including cancer. At the 12 months of follow-up period, approximately 75% of symptom improvement was noticed in 63% of patients and approximately 50% of symptom improvement was documented in 91% of patients.

This is the first study that involves a large number of human patients. The amount of adipose tissue varies: 20–90 g. Thus, the estimated number of ADSCs injected may range from 100,000 to 18,000,000 cells. Further, no PRP nor HA was used. However, the results are encouraging.

## 5. Discussions 

Adipose tissue is considered to be a preferable source of MSCs due to its ease of accessibility and the availability of a large number of stem cells per gram of adipose tissue. In adipose tissue, 1% to 10% of nucleated cells are considered to be ADSCs whereas only 0.0001–0.01% of nucleated cells in the bone marrow are stem cells [14]. In addition, the number of nucleated cells in adipose SVF can range from 500,000 to 2,000,000 cells per gram of adipose tissue [40]. The range of MSCs in 1 g of adipose tissue may be 5,000–200,000 stem cells [40]. Thus, theoretically, 0.5–20 million ADSCs can be extracted from 100 g of adipose tissue. If the number of MSCs in adipose SVF is 5%, approximately 10 million ADSCs can be obtained from 100 g of adipose tissue.

ADSCs, as one specific form of MSCs, have been shown to regenerate cartilage in animals [15, 57, 58]. However, some authors claim adipose SVF alone may not be sufficient to regenerate cartilage in animals [18]. Interestingly, in this review, 11 of thirteen human studies had used autologous PRP in addition to ADSCs in the form of SVF.

Autologous PRP may play an important role in cartilage regeneration. PRP releases a variety of growth factors when activated. Centeno et al. used platelet lysate to grow MSCs that were injected into a human knee for cartilage regeneration [11]. The TGF-*β* contained in PRP may be necessary for differentiation of MSCs into cartilage cells [43].

Autologous PRP may also play a role as a scaffold, influencing stem cell adherence to lesions, as well as stem cell growth and differentiation. When properly activated, autologous PRP can become a “curd-like” substance and can thus operate as scaffold, as shown by Kim et al. [49]. Although autologous PRP alone may not regenerate cartilage as shown by Koh et al. [55], PRP may enhance ADSCs in SVF to adhere to the cartilage lesion and proliferate.

The randomized, double-blind dose escalation clinical study reported by Jo et al. clearly showed the likelihood of cartilage regeneration with ADSCs alone without any additives such as PRP or HA [21]. In the study, Jo et al. showed a direct relationship between the number of stem cells injected and the amount of cartilage regenerated. The amount of cartilage regenerated was much greater with 100 million ADSCs than 50 million ADSCs injected. This was documented by arthroscopies and MRIs [21].

On the other hand, the study by Michalek et al. did not use any other additives although the numbers of ADSCs injected are estimated to be less than the number of ADSCs used in the study by Jo et al. Also, the study by Michalek et al. did not use PRP or HA. Among all the studies reviewed in this paper, Michalek et al. study is the only one that did not have any visible objective data, such as MRI or arthroscopic photos, although significant clinical improvement has been documented.

Although most of the studies in this review used a relatively large volume (approximately 100 g) of adipose tissue, three studies used a relatively small volume (approximately 20 g) of adipose tissue. However, these three studies used PRP with low amount of adipose tissue and showed clinical improvement in patients. Therefore, it can only be estimated that adipose tissue from different regions of patients' abdomens may contain different number of stem cells.

It has been shown that different individuals have different density in the adipose tissue, indicating different amount of matrix [59]. ADSCs exist within matrix of adipose tissue around the blood vessels. Consequently, it can be concluded that higher density of adipose tissue may contain higher density of matrix and thus yields higher number of stem cells. Furthermore, the method of liposuction may affect the results of ADSCs yield in the SVF. Compared to surgical resection of adipose tissue, liposuction has been shown to produce higher percentage of viable cells in lipoaspirates [60].

In addition to differences in adipose tissue and its extraction, the concentration and incubation time of collagenase are other important factors affecting the yield of ADSCs and their viability in SVF. Since high dosage or exposure to collagenase may be toxic to ADSCs, excess amount of collagenase can decrease the ADSC viability while insufficient amount of collagenase may result in inefficient and inadequate amount of ADSC yield [61].

Based on the study by Jo et al., it is logical to expect higher rates of improvement with a higher amount of ADSCs obtained and used for cartilage regeneration. However, the direct dose relationship was not clearly observed when comparing the 12 studies that involved ADSCs in the form of SVF. This may be due to variability in adipose SVF obtained from different individuals, stem cell viability when processing adipose tissue and injecting SVF, stem cell adherence, and stem cell growth. Also addition of growth factors, such as PRP, and scaffold material, such as HA, may be important as shown by Koh et al. [49, 55]. Dregalla et al. showed that local anesthetics can also have very significant negative effects on stem cell survival and adherence [62].

Another factor can be the scaffolds themselves. HA works as a scaffold [50], and the studies [20, 44, 47, 48] reported by Pak et al. used HA for such purposes. Adipose SVF contains a variety of cell types including ADSCs and extracellular matrix (ECM) [13, 14]. Such ECM contained in the adipose SVF may also work as scaffold and assist ADSCs to adhere to the lesion, proliferate, and differentiate [51]. ECM also may excrete a variety of cytokines and growth factors, affecting the cartilage regeneration by MSCs [51, 63–65].

The mode of injection does not seem be a major determining factor in cartilage regeneration. Most studies reported by Koh et al. used intra-articular injections of adipose SVF under arthroscopic guidance. However, it is unclear whether such an injection is better than a percutaneous injection. Arthroscopic examination of knees requires spinal or general anesthesia; thus, it is not considered to be a minimally invasive procedure. In addition, arthroscopic lavage and debridement for OA of the knee are ineffective [66]. A head-to-head study may be necessary to determine if such an invasive procedure outweighs the efficacy of percutaneous injections.

## 6. Conclusions 

At present, there is no cure for painful OA in stages 2 and 3. For these patients, the intra-articular injection of ADSCs in the form of SVF can be an alternative treatment for now. As described in this review, the joint injection of ADSCs in the form of SVF with PRP can be safe and efficacious. Moreover, obtaining approximately 100 g of adipose tissue and percutaneous joint injections is considered to be a minimally invasive procedure and can be readily accepted by patients. These procedures carry relatively low rates of morbidity and side effects.

Although a large amount of injecting ADSCs is more efficacious in regenerating cartilage, the studies reviewed in this paper have shown that ADSCs in the form of SVF with PRP can be efficacious in symptom improvement.

However, lack of well-designed studies with control on using different methods and components of the injections still leaves many questions unanswered. In addition, the lack of understanding of the mechanism of action of ADSCs dictates the need for more clinical trials.

## Figures and Tables

**Table 1:** Clinical studies on ADSC treatments for cartilage defects.

Study (yr)	Intervention treatment	Study type	Number of subjects	Subject characteristic [age (yr); gender]	Previous therapy	Concurrent treatment	Follow-up (mo)	Outcome measures	Results	Authors' conclusion
Pak (2011) [20]	Adipose SVF (ADSC) + PRP via percutaneous injections	Case report	2	70 and 79; 2 F with chronic knee pain	Various treatments without any success	None	3	VAS; functions (FRI, ROM); MRI	VAS/function improvements and MRI evidence of cartilage regeneration	ADSC + PRP: potentially effective in regenerating cartilage in humans

Koh and Choi (2012) [52]	Adipose SVF (ADSC) + PRP via percutaneous injections	Nonrandomized, retrospective, comparative study: ADSC + PRP versus PRP alone	25 Study group (ADSC + PRP): 25; control group (PRP alone): 25	Study group: mean 54.1 (range, 34–69); 8 M and 17 F	Various treatments without any success	None	16.4	VAS; functions (Lysholm, Tegner)	ADSC + PRP: more effective than PRP-control group	ADSC + PRP: potentially effective in patients with cartilage defects

Pak et al. (2013) [44]	Adipose SVF (ADSC) + PRP via percutaneous injections	Retrospective cohort study	91	Mean 51.23 ± 1.50 (range, 18–78); 45 M and 46 F	Various treatments without any success	None	26.62 ± 0.32	VAS; functions	Statistically significant improvement in both VAS and functions	ADSC + PRP: safe and potentially effective

Pak et al. (2013) [47]	Adipose SVF (ADSC) + PRP via percutaneous injections	Case series	3	43 and 63; 2 F 54; 1 M All with chronic knee pain	Various treatments without any success	None	3	VAS; functions (FRI, ROM); MRI	VAS/function improvements and MRI evidence of cartilage regeneration	ADSC + PRP: effective in treating chondromalacia patellae patients

Koh et al. (2013) [53]	Adipose SVF (ADSC) + PRP via percutaneous injection	Case series	18	Mean 54.6; 6 M and 12 F	Various treatments without any success	Arthroscopic lavage before knee-fat-pad-derived adipose SVF + PRP injection	24.3	VAS; functions (WOMAC, Lysholm); MRI	VAS/function/MRI improvements	ADSC + PRP: effective in treating OA of knees

Koh et al. (2015) [22]	Adipose SVF (ADSC) + PRP under arthroscopic guidance	Case series	30 for adipose SVF + PRP injection; 16 for second-look arthroscopy	Mean 70.3 (range, 65–80); 5 M and 25 F	Various treatments without any success	Arthroscopic lavage before ADSCs + PRP injection	24	VAS; functions; 2nd-look arthroscopy	VAS/function improvements; improved and maintained cartilage status	ADSCs + PRP: effective in treating elderly patients with OA

Pak et al. (2014) [48]	Adipose SVF (ADSC) + PRP via percutaneous injections	Case report	1	32; 1 F with chronic knee pain due to meniscus tear	Various treatments without any success	None	3	VAS; functions (FRI, ROM); MRI	VAS/function improvements and MRI evidence of cartilage regeneration	ADSC + PRP: effective in treating cartilage defect lesions, including meniscus tear

Bui et al. (2014) [54]	Adipose SVF (ADSC) + PRP via percutaneous injections	Case series	21	>18; ND	Various treatments without any success	None	8.5	VAS; functions; MRI	VAS/function/MRI improvements	ADSC + PRP: effective in treating OA of knees

Jo et al. (2014) [21]	Culture-expanded ADSC via arthroscopic injections	Randomized double-blind dose escalation study (a proof-of-concept clinical trial)	18	61–65; 3 M and 15 F	Various treatments without any success	None	6	VAS; functions; MRI; arthroscopy; histology	VAS/function/MRI/ arthroscopic/histological improvements	1.0 × 10^8^ ADSCs into the osteoarthritic knee improved function and pain of the knee joint. Radiological, arthroscopic, and histological measures demonstrated regeneration of hyaline-like articular cartilage

Koh et al. (2014) [23]	Adipose SVF (ADSC) + PRP under arthroscopic guidance	Case series	35 with 37 knee joints	Mean 57.4 (range, 48–69); 14 M and 21 F	Various treatments without any success	Arthroscopic lavage before adipose SVF + PRP injection	12.7	VAS; functions; arthroscopy	94% patients had excellent clinical improvement; 76% had abnormal repair tissue	Scaffolds may be needed to treat patients with large cartilage lesions

Koh et al. (2014) [55]	Adipose SVF (ADSC) + PRP under arthroscopic guidance	Comparative study: adipose SVF + PRP versus PRP only	44	ND	Various treatments without any success	Open-wedge high tibial osteotomy	24	VAS; functions; arthroscopy	Adipose SVF + PRP is more effective than PRP alone	ADSC therapy, in conjunction with HTO, mildly improved cartilage healing and showed good clinical results compared with PRP only

Kim et al. (2015) [49]	Adipose SVF (ADSC) under arthroscopic guidance	Comparative study: adipose SVF versus adipose SVF + fibrin glue (as a scaffold)	54	Mean 57.5 ± 5.8; 22 M and 32 F	Various treatments without any success	None	28.6	VAS; functions; arthroscopy	No significant difference	Clinical and arthroscopic outcomes of ADSC implantation were encouraging for OA knees in both groups, although there were no significant differences in outcome scores between groups

Michalek et al. (2015) [56]	Adipose SVF (ADSC) via percutaneous injection	Multicenter case control study	1,114	Median 62 (range, 19–94); 589 M and 525 F	Various treatments without any success	None	Median 17.2	VAS; functions	VAS/function improvements	Adipose SVF is a novel and promising treatment approach for patients with degenerative OA. ADSC is safe and cost-effective

SVF: stromal vascular fraction; ADSC: adipose tissue-derived stem cells; PRP: platelet rich plasma; OA: osteoarthritis; yr: year; mo: month; M: male; F: female; ND: not described; HTO: high tibial osteotomy; VAS: visual analogue scale; FRI: functional rate index; ROM: range of motion; WOMAC: Western Ontario and McMaster Universities osteoarthritis index; Lysholm: Lysholm scores; Tegner: Tegner activity scale.
